# Identification of BMI-related high-risk feature combinations for diabetes among young adults with normal baseline fasting plasma glucose using interpretable machine learning: a health check-up cohort study

**DOI:** 10.3389/fendo.2026.1850071

**Published:** 2026-05-15

**Authors:** Zhen Xu, Ying Zhang, Huachun Zhang

**Affiliations:** Nursing Department, Longhua Hospital Affiliated to Shanghai University of Traditional Chinese Medicine, Shanghai, China

**Keywords:** body mass index, diabetes, interpretable machine learning, normal fasting plasma glucose, SHAP, young adults

## Abstract

Body mass index (BMI) is an easily obtainable indicator for diabetes risk screening, but its residual risk value among young adults with normal fasting plasma glucose (FPG) remains insufficiently understood. This cohort study investigated the association between BMI and incident diabetes, its nonlinear risk pattern, and BMI-related risk structures among young adults with normal baseline FPG. Data were obtained from the Rich Healthcare Group health check-up database in China. Participants aged <40 years without diabetes at baseline, with complete BMI data and at least one follow-up visit, were included; those with baseline FPG <5.6 mmol/L were defined as the primary analytic population. Cox regression and restricted cubic spline analysis were used to examine the association between BMI and incident diabetes. Four machine learning models were compared, with logistic regression selected as the primary interpretable model and XGBoost used as an exploratory nonlinear model. SHapley Additive exPlanations were applied to interpret model-derived variable contributions. A total of 103,693 participants were included, and 266 incident diabetes events occurred during a median follow-up of 2.99 years. BMI was independently associated with incident diabetes in the multivariable Cox model (HR = 1.284, 95% CI: 1.250–1.319; P <0.001). Restricted cubic spline analysis showed a significant nonlinear association, with risk increasing more steeply beyond approximately 28 kg/m². In the validation set, logistic regression and XGBoost achieved ROC-AUC values of 0.812 and 0.817, respectively; however, their low PR-AUC values indicated limited ability to identify true positive cases under the very low event rate. SHAP analysis identified BMI as the most influential predictor in the exploratory XGBoost model and suggested possible model-derived joint contribution patterns involving triglycerides and systolic blood pressure, but formal Cox-based interaction testing did not confirm statistically significant multiplicative interactions. These findings suggest that BMI-related diabetes risk among normoglycemic young adults is nonlinear and embedded within a broader metabolic risk structure. Combining conventional regression with interpretable machine learning may support earlier identification and refined risk stratification of young adults at increased diabetes risk before fasting glucose becomes abnormal.

## 1 Introduction

In recent years, the burden of diabetes among young people has increased substantially worldwide, making early risk identification an important public health priority. Trend analyses from the Global Burden of Disease 2021 study showed a marked rise in type 2 diabetes incidence among individuals aged 10–24 years, while IDF-based evidence also indicates a sustained increase among adults aged 20–39 years (1, 2). Early-onset type 2 diabetes is associated with higher risks of mortality and vascular complications, and cumulative exposure to prediabetes and metabolic syndrome components during youth has been linked to a higher future risk of diabetes (3–5).

Given that baseline fasting plasma glucose is a strong predictor of incident diabetes, this study focused on young adults with normal baseline fasting plasma glucose to identify early residual risk signals before abnormal fasting glucose develops (6). Among candidate indicators, body mass index is an important clue for risk identification, but it is insufficient on its own for refined stratification. In East Asian populations, diabetes often occurs at relatively low body mass index levels, and previous studies have shown that body mass index alone cannot reliably distinguish truly low-risk individuals (7–10).

Traditional Cox and logistic regression models are useful for estimating average associations, but they may be limited in capturing nonlinear relationships, higher-order interactions, and latent heterogeneous risk structures (11). Interpretable machine learning can complement conventional regression by better characterizing complex risk patterns and explaining variable contributions through tools such as SHAP (12, 13). Accordingly, this study aimed to investigate the association between body mass index and incident diabetes among young adults with normal baseline fasting plasma glucose and to identify body mass index-related high-risk feature combinations. Specifically, we combined Cox proportional hazards regression, restricted cubic spline analysis, multiple machine learning algorithms, and SHAP interpretation to evaluate both conventional statistical associations and model-derived nonlinear risk patterns. This analytical framework showed that body mass index was independently associated with incident diabetes, that its risk effect increased nonlinearly at higher body mass index levels, and that logistic regression and extreme gradient boosting demonstrated stable validation performance for 3-year diabetes risk prediction. These findings support the integration of conventional regression and interpretable machine learning for early diabetes risk identification and refined risk stratification among normoglycemic young adults.

## 2 Materials and methods

### 2.1 Study design and data source

The data used in this study were extracted from a computerized database established by Rich Healthcare Group in China, which contains medical records of all participants who underwent health examinations between 2010 and 2016. We included participants who were free of diabetes at baseline, aged <40 years, had complete BMI data, and had completed at least one follow-up visit (n = 11609). The exclusion criteria were as follows: baseline FPG >5.6 mmol/L (n = 7910); incomplete information on age, sex, family history, or lifestyle (n = 5); and incomplete outcome information (n = 1). Participants were excluded if they had baseline FPG ≥5.6 mmol/L, missing outcome information, or missing core eligibility variables required for cohort construction. The 5 participants excluded for incomplete covariate data had missing information in core demographic or baseline variables required for defining the analytic cohort. Missingness in all candidate predictors used for model development was further assessed and is reported in **Table 1**.

**Table 1:** Missingness for variables before imputation.

Variable	Missing, n	Missing, %
Follow-up time	0	0.00
Incident diabetes outcome status	0	0.00
Age	0	0.00
Sex	0	0.00
BMI	0	0.00
Baseline FPG	0	0.00
Systolic blood pressure	0	0.00
Diastolic blood pressure	0	0.00
Total cholesterol	2743	2.65
Triglycerides	2763	2.66

A total of 103,693 participants were ultimately included in the present analysis, including 55,402 men and 48,291 women. Cohort entry was defined as the date of the first visit.

### 2.2 Outcome definition

The outcome was incident diabetes during follow-up, defined as self-reported diabetes or newly detected diabetes with FPG >7 mmol/L. Participants were censored at the date of diabetes diagnosis or at the last follow-up visit, whichever occurred first.

### 2.3 Candidate predictors

The candidate predictors included age, sex, BMI, total cholesterol (TC), triglycerides (TG), low-density lipoprotein cholesterol (LDL-C), high-density lipoprotein cholesterol (HDL-C), systolic blood pressure (SBP), diastolic blood pressure (DBP), and family history of diabetes.

### 2.4 Statistical modeling and machine learning procedures

Given the strong predictive value of baseline FPG for incident diabetes, the primary analysis was restricted to young adults with normal baseline FPG (FPG <5.6 mmol/L) to reduce the dominant influence of individuals who had already entered the prediabetic range on risk identification. This cutoff was defined according to the ADA criteria for normal fasting glucose and impaired fasting glucose (14). To examine the robustness of the findings, a sensitivity analysis was further conducted using FPG <6.1 mmol/L as an alternative cutoff. In addition, in the machine learning analysis, a primary model excluding FPG was first constructed to identify BMI-related residual risk structures beyond glycemia itself; an extended model including FPG was then developed to assess the stability of the main findings.

Missingness was assessed for all variables used in the Cox regression and machine learning analyses. Core variables required for cohort construction and outcome definition were required to be complete, and participants with missing core variables were excluded. For non-core candidate predictors with missing values, imputation was performed within the training set only. Continuous variables were imputed using training-set medians, and categorical variables were imputed using training-set modes; the same parameters were then applied to the validation set.

First, Cox proportional hazards regression was used to analyze the association between BMI and incident diabetes, with hazard ratios (HRs) and 95% confidence intervals (CIs) calculated. Restricted cubic splines (RCS) were further applied to examine whether a nonlinear association existed between BMI and diabetes risk.

In the machine learning analysis, the primary goal was not merely to maximize predictive accuracy, but to identify BMI-related residual risk structures and potential high-risk feature combinations. Because the present study used structured tabular data and aimed simultaneously to perform risk prediction, identify key features, and provide interpretable results, logistic regression (LR), classification and regression tree (CART), random forest (RF), and extreme gradient boosting (XGBoost) were selected as candidate models. The primary machine learning model excluded FPG so that early BMI-related risk features beyond blood glucose itself could be captured; an extended model including FPG was then built to test the robustness of the principal findings. LR was selected as the primary interpretable model because it provides transparent coefficients, stable validation performance, and relatively straightforward clinical interpretation. However, LR mainly captures additive and approximately linear effects unless nonlinear terms are explicitly modeled. Therefore, XGBoost was retained as an exploratory nonlinear model to complement LR by capturing potential nonlinear structures and higher-order predictor patterns. The two models were thus used for different purposes: LR for stable and interpretable short-term risk stratification, and XGBoost for exploratory pattern discovery.

The dataset was randomly split into training and validation sets using stratification by outcome. To avoid information leakage, missing-value imputation, variable encoding, and all other preprocessing steps were performed only within the training set, and the same parameters were then applied to the validation set. Because the 3-year incidence of diabetes was low in this cohort, model performance was evaluated primarily using the precision–recall area under the curve (PR-AUC), together with the area under the receiver operating characteristic curve (AUC), sensitivity, specificity, positive predictive value (PPV), negative predictive value (NPV), Brier score, and calibration curves.

Based on the overall performance evaluation, LR was selected as the main interpretable model to provide stable and directly interpretable risk estimates. Because XGBoost is more capable of capturing complex nonlinear relationships and variable interactions, it was further used as an exploratory secondary model. SHapley Additive exPlanations (SHAP) were used to interpret variable contributions in the exploratory XGBoost model. SHAP dependence and interaction visualizations were used to explore model-derived joint contribution patterns between BMI and other key variables. These visualizations were not interpreted as formal statistical tests of interaction. Formal interaction assessment was subsequently performed using Cox regression with prespecified threshold-based interaction terms. On this basis, clustering analysis was further planned to construct latent high-risk phenotypes in young adults.

### 2.5 Algorithmic representation of the analytical workflow

The analysis was conducted according to a predefined workflow that included cohort construction, outcome definition, predictor extraction, conventional statistical modeling, machine learning model development, model interpretation, and formal testing of BMI-related high-risk combinations: 1) eligible participants were selected from the health check-up cohort according to predefined inclusion and exclusion criteria, and young adults with baseline FPG <5.6 mmol/L were defined as the primary analytic population. 2) incident diabetes during follow-up was defined as self-reported diabetes or newly detected diabetes with FPG >7 mmol/L, and participants were censored at the date of diabetes diagnosis or the last follow-up visit. 3) candidate predictors, including age, sex, BMI, blood pressure indicators, lipid indicators, family history of diabetes, and lifestyle factors, were extracted for analysis. 4) Cox proportional hazards regression was used to estimate the association between BMI and incident diabetes, and restricted cubic spline analysis was performed to examine the nonlinear risk pattern of BMI. 5) the dataset was divided into training and validation sets using outcome-stratified sampling, and four candidate machine learning models, including logistic regression, classification and regression tree, random forest, and extreme gradient boosting, were developed without baseline FPG in the primary model. 6) model performance was evaluated using AUC, PR-AUC, sensitivity, specificity, positive predictive value, negative predictive value, Brier score, and calibration curves. Logistic regression was selected as the primary interpretable model because of its stable validation performance and clinical interpretability, whereas extreme gradient boosting was used as an exploratory nonlinear model because of its ability to capture complex nonlinear patterns. Finally, SHAP analysis was applied to interpret variable contributions and identify BMI-related joint risk patterns, followed by Cox interaction testing to formally examine threshold-based high-risk combinations involving BMI ≥28 kg/m², elevated triglycerides, elevated systolic blood pressure, and family history of diabetes.

### 2.6 Statistical analysis

Baseline characteristics were described according to variable type. Continuous variables with an approximately normal distribution are presented as mean ± standard deviation (SD), skewed continuous variables as median and interquartile range (IQR), and categorical variables as frequency (percentage). Baseline characteristics were compared between participants who developed incident diabetes during follow-up and those who did not. Continuous variables were compared using the independent-samples t test or Mann–Whitney U test as appropriate according to their distribution, and categorical variables were compared using the chi-square test or Fisher’s exact test. All statistical tests were two-sided, and P <0.05 was considered statistically significant.

Data preprocessing and machine learning analyses were performed using Python 3.14.3, primarily with the pandas, numpy, matplotlib, scikit-learn, xgboost, lightgbm, and shap packages. Cox regression and restricted cubic spline analyses were conducted using R version 4.5.3. All statistical analyses were two-sided, and P <0.05 was considered statistically significant.

## 3 Results

### 3.1 Baseline characteristics

A total of 103,693 participants were finally included in the analysis. The median follow-up duration was 2.99 years. During follow-up, 266 participants developed incident diabetes, accounting for 0.26% of the total sample, whereas the remaining 103,427 participants did not develop diabetes. The mean age of the participants was 32.40 ± 3.86 years; 53.43% were male and 46.57% were female. At baseline, mean BMI was 22.49 ± 3.38 kg/m², mean SBP was 115.16 ± 13.84 mmHg, mean DBP was 71.55 ± 9.61 mmHg, mean FPG was 4.72 ± 0.49 mmol/L, mean total cholesterol was 4.48 ± 0.82 mmol/L, mean triglycerides were 1.14 ± 0.83 mmol/L, and 1.83% of participants had a positive family history of diabetes. Before imputation, missingness was observed only for total cholesterol and triglycerides among the variables included in the final Cox regression and machine learning analyses, with missing rates of 2.65% and 2.66%, respectively. The baseline characteristics of the training and validation sets are shown in **Table 2**.

**Table 2:** Baseline characteristics of the training and validation sets.

Characteristic	Training set	Validation set	P value
Participants	82954	20739	—
Incident diabetes			0.553
No	82796 (99.81%)	20706 (99.84%)	
Yes	158 (0.19%)	25 (0.16%)	
Age (years)	32.81 ± 3.68	32.84 ± 3.67	0.438
Sex			0.734
Male	44768 (54.07%)	11255 (54.27%)	
Female	38186 (45.93%)	9484 (45.73%)	
BMI (kg/m²)	22.44 ± 3.35	22.44 ± 3.36	0.920
SBP (mmHg)	115.15 ± 13.82	115.16 ± 13.86	0.933
DBP (mmHg)	71.77 ± 9.56	71.77 ± 9.47	0.986
FPG (mmol/L)	4.62 ± 0.52	4.63 ± 0.52	0.608
Total cholesterol (mmol/L)	4.48 ± 0.82	4.48 ± 0.82	0.779
Triglycerides (mmol/L)	1.13 ± 0.83	1.11 ± 0.78	0.244
Family history of diabetes			0.112
No	81070 (97.73%)	20324 (98.00%)	
Yes	1884 (2.27%)	415 (2.00%)	

### 3.2 Association between BMI and incident diabetes

**Figure 1:**
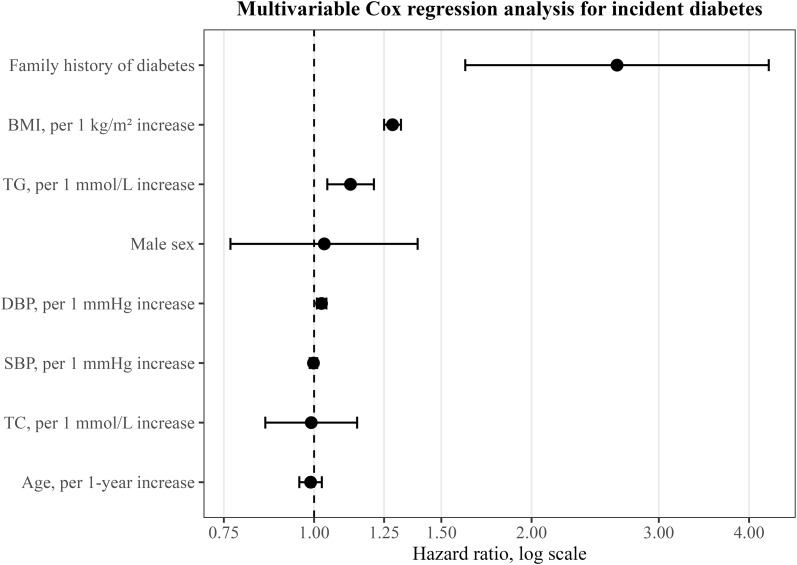
Forest plot of multivariable Cox regression analysis for incident diabetes.

In the primary analysis population with baseline FPG <5.6 mmol/L, univariable Cox proportional hazards regression showed that BMI, age, male sex, SBP, DBP, total cholesterol, triglycerides, and family history of diabetes were all associated with the risk of incident diabetes **Figure 1**, **Table 3**. After further adjustment for age, sex, SBP, DBP, total cholesterol, triglycerides, and family history of diabetes, BMI remained independently associated with incident diabetes (HR = 1.284, 95% CI: 1.250–1.319, P <0.001), indicating that each 1 kg/m² increase in BMI was associated with a 28.4% increase in the risk of incident diabetes. In addition, DBP (HR = 1.024, 95% CI: 1.009–1.040, P = 0.002), triglycerides (HR = 1.123, 95% CI: 1.043–1.210, P = 0.002), and family history of diabetes (HR = 2.626, 95% CI: 1.619–4.259, P <0.001) were also independently associated with incident diabetes, whereas age, sex, SBP, and total cholesterol were not statistically significant in the multivariable model.

**Table 3:** Univariable and multivariable Cox proportional hazards regression analyses of incident diabetes among participants with baseline FPG <5.6 mmol/L.

Variable	Univariable HR (95% CI)	P value	Multivariable HR (95% CI)	P value
BMI, per 1 kg/m² increase	1.315 (1.286–1.345)	<0.001	1.284 (1.250–1.319)	<0.001
Age, per 1-year increase	1.038 (1.003–1.074)	0.033	0.989 (0.954–1.025)	0.538
Male sex	2.494 (1.896–3.281)	<0.001	1.033 (0.766–1.391)	0.833
SBP, per 1 mmHg increase	1.042 (1.034–1.050)	<0.001	0.998 (0.986–1.010)	0.697
DBP, per 1 mmHg increase	1.060 (1.049–1.070)	<0.001	1.024 (1.009–1.040)	0.002
TC, per 1 mmol/L increase	1.426 (1.260–1.614)	<0.001	0.991 (0.856–1.147)	0.906
TG, per 1 mmol/L increase	1.277 (1.233–1.322)	<0.001	1.123 (1.043–1.210)	0.002
Family history of diabetes	3.001 (1.859–4.844)	<0.001	2.626 (1.619–4.259)	<0.001

### 3.3 Nonlinear association between BMI and incident diabetes risk

**Figure 2:**
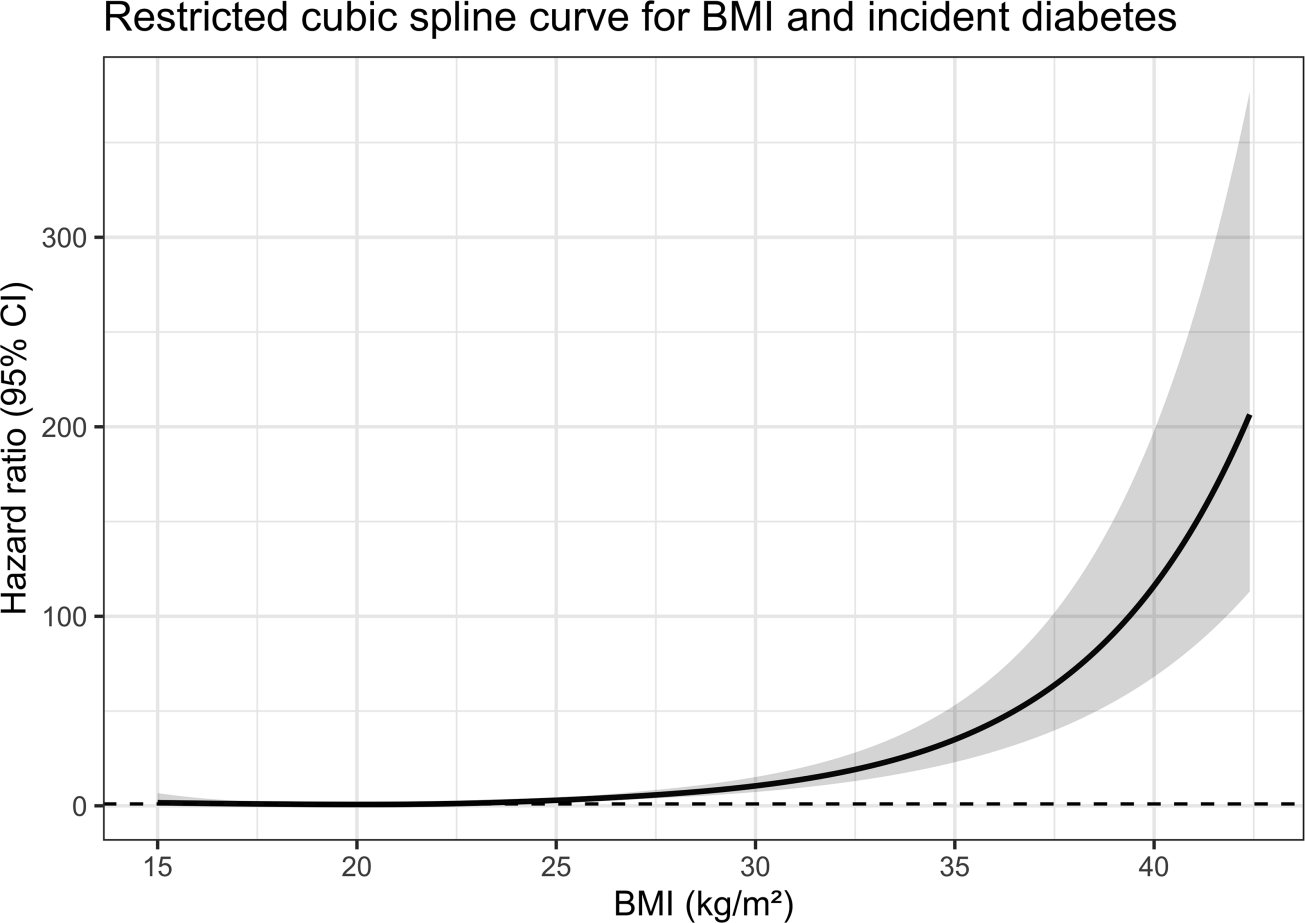
Restricted cubic spline curve for the association between BMI and incident diabetes risk in participants with baseline FPG <5.6 mmol/L.

Restricted cubic spline analysis showed that BMI was significantly associated with the risk of incident diabetes (overall P <0.001) and that this association was nonlinear (nonlinear P = 0.008). At lower BMI levels, the change in risk was relatively modest; however, as BMI increased, the risk gradually rose and became markedly steeper after approximately 28 kg/m², suggesting that this range may represent a critical threshold for accelerated risk increase (**Figure 2**). These findings indicate that the effect of increasing BMI on incident diabetes is not uniform but rather becomes stronger at higher BMI levels. Together with the SHAP dependence plot and commonly used clinical obesity cutoffs, BMI ≥28 kg/m² was used in subsequent analyses to define high BMI.

### 3.4 Machine learning model selection

**Figure 3:**
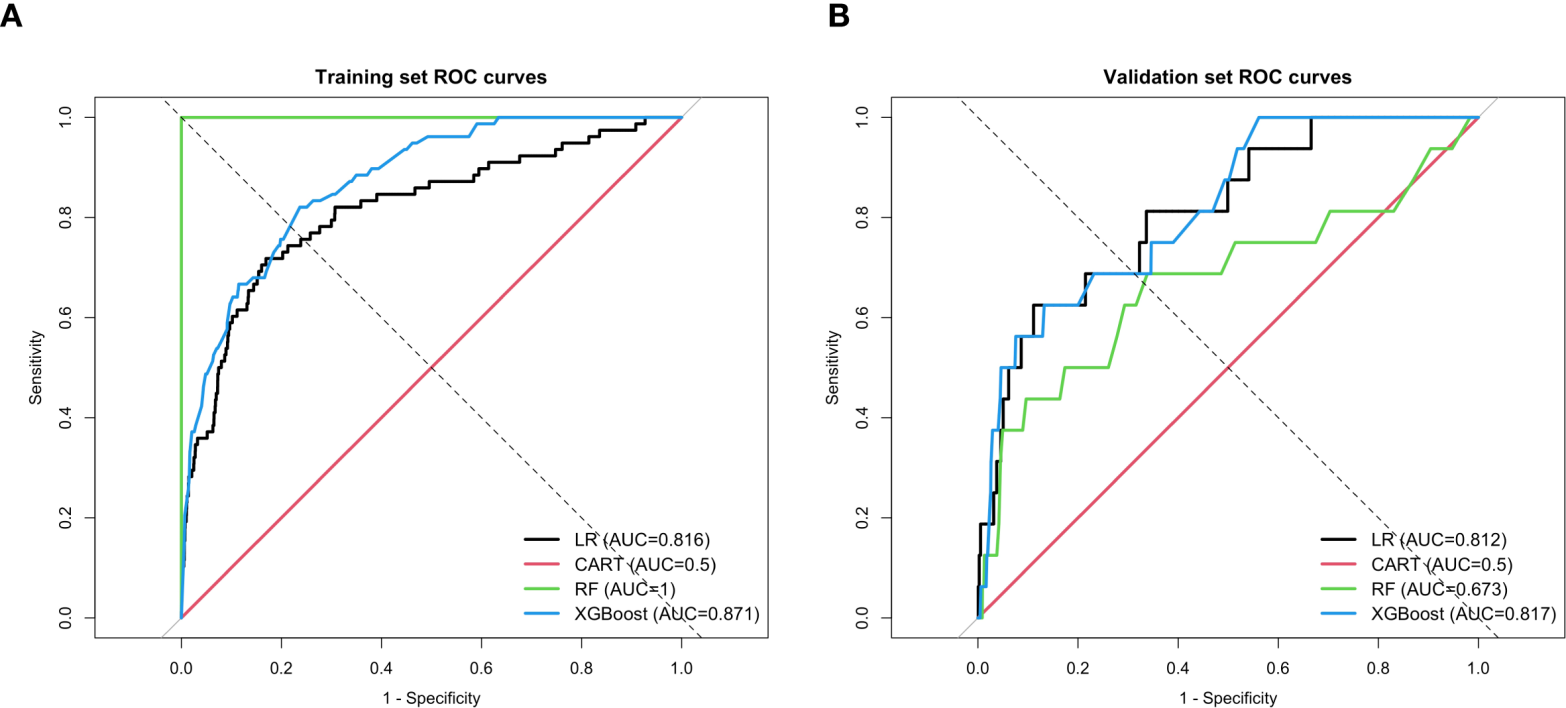
**(A)** ROC curves of machine learning models in the training set. **(B)** ROC curves of machine learning models in the validation set.

In the primary analysis population with baseline FPG <5.6 mmol/L, 3-year incident diabetes prediction models excluding FPG were developed using LR, CART, RF, and XGBoost. The ROC curves shown in **Figure 3A, B** indicate that LR and XGBoost exhibited relatively good discrimination in both the training and validation sets. Specifically, the AUCs of LR were 0.816 and 0.812 in the training and validation sets, respectively, whereas those of XGBoost were 0.871 and 0.817. In contrast, RF yielded AUCs of 1.000 and 0.673 in the training and validation sets, respectively, suggesting excellent apparent performance in the training set but limited generalizability. CART showed an AUC of 0.500 in both datasets, indicating poor discriminative ability. Although XGBoost achieved a slightly higher validation ROC-AUC than LR, the difference was small. Moreover, XGBoost did not show a clear advantage in PR-AUC under the low-event-rate setting. Considering the clinical interpretability of LR, its stable validation performance, and the primary aim of constructing an interpretable short-term risk stratification model, LR was retained as the primary model. XGBoost was used as an exploratory complementary model to assess nonlinear contribution patterns and potential joint predictor structures.

Based on these results, LR and XGBoost were retained for further interpretation because they showed relatively stable validation performance and represented complementary modeling strategies: a clinically interpretable conventional model and an ensemble learning model capable of capturing nonlinear structures. CART yielded an AUC of 0.500 in both the training and validation sets, indicating almost no meaningful discriminative ability. RF achieved an AUC of 1.000 in the training set, but its validation AUC decreased to 0.673, suggesting a clear risk of overfitting. Therefore, LR was used as the primary interpretable model in the subsequent analysis, whereas XGBoost was used as the exploratory nonlinear model and further interpreted using SHAP to identify BMI-related variable contributions and potential joint risk patterns.

### 3.5 SHAP interpretation analysis

**Figure 4:**
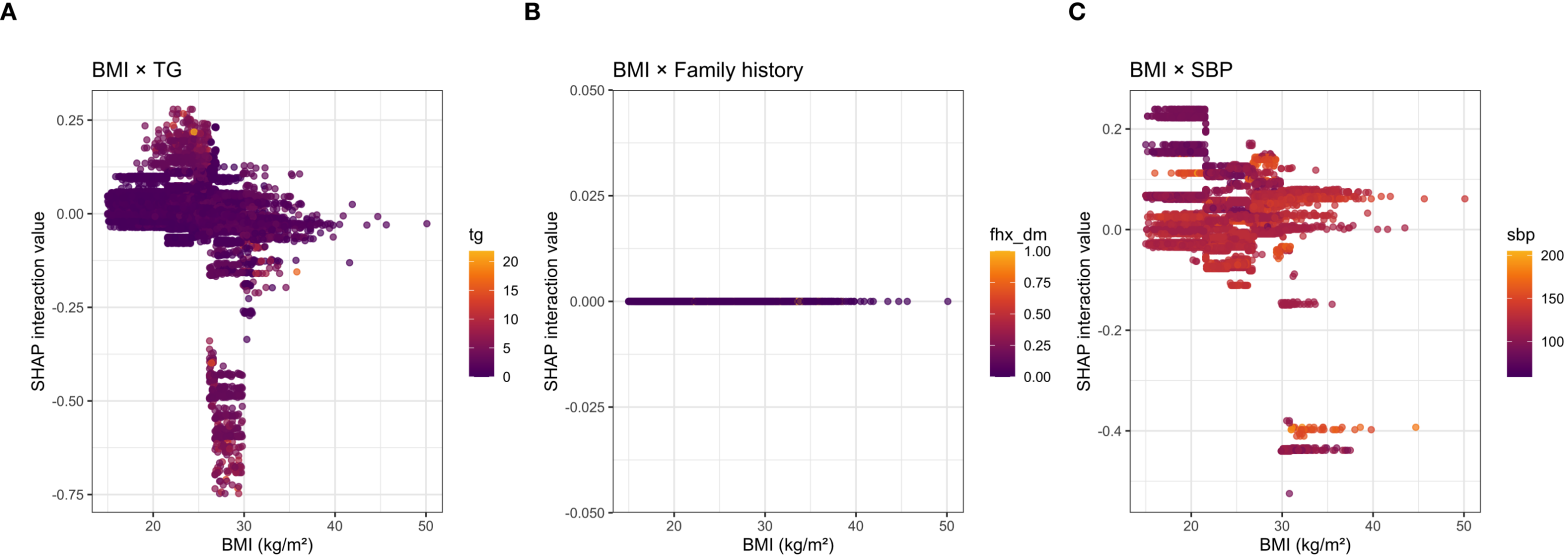
SHAP interaction visualization between BMI and key variables. **(A)** SHAP interaction visualization between BMI and triglycerides (TG). **(B)** SHAP interaction visualization between BMI and family history of diabetes. **(C)** SHAP interaction visualization between BMI and systolic blood pressure (SBP).

**Figure 5:**
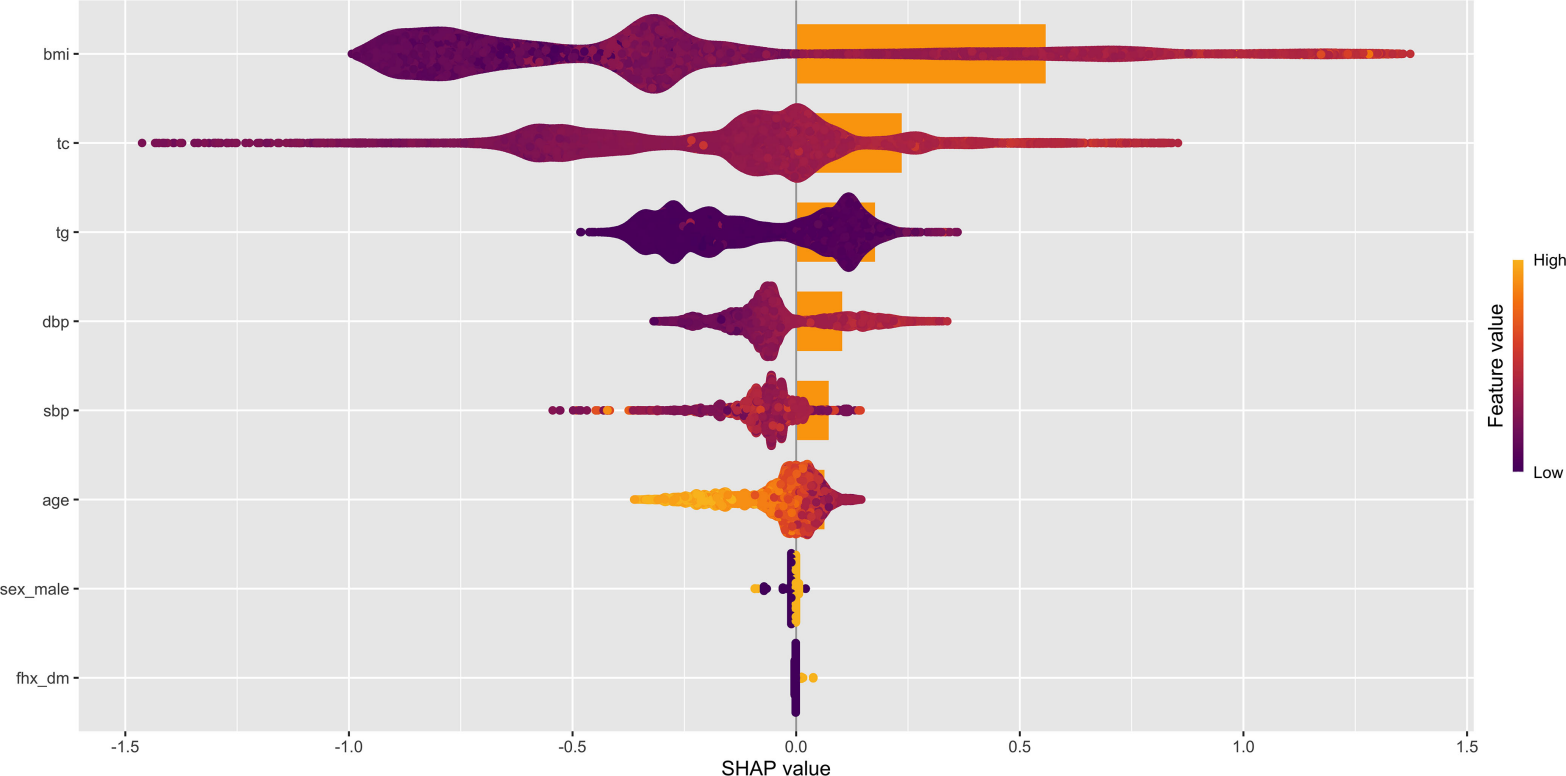
SHAP variable importance plot and summary beeswarm plot for the exploratory XGBoost model.

SHAP analysis showed that (**Figure 4**), in the exploratory XGBoost model excluding FPG, BMI was the variable contributing the most to the model output (**Figure 5**), followed by total cholesterol, triglycerides, DBP, and SBP, suggesting that body weight status and its related metabolic features play an important role in identifying 3-year diabetes risk even at the normoglycemic stage (**Figure 4**). From the distribution of SHAP values, observations with higher BMI were more often located in the positive SHAP-value region, whereas observations with lower BMI were more often located in the negative SHAP-value region, indicating that higher BMI tended to push the model output toward a higher-risk direction, whereas lower BMI tended to push it toward a lower-risk direction. This finding was consistent with the results of the Cox regression and RCS analyses.

**Figure 6:**
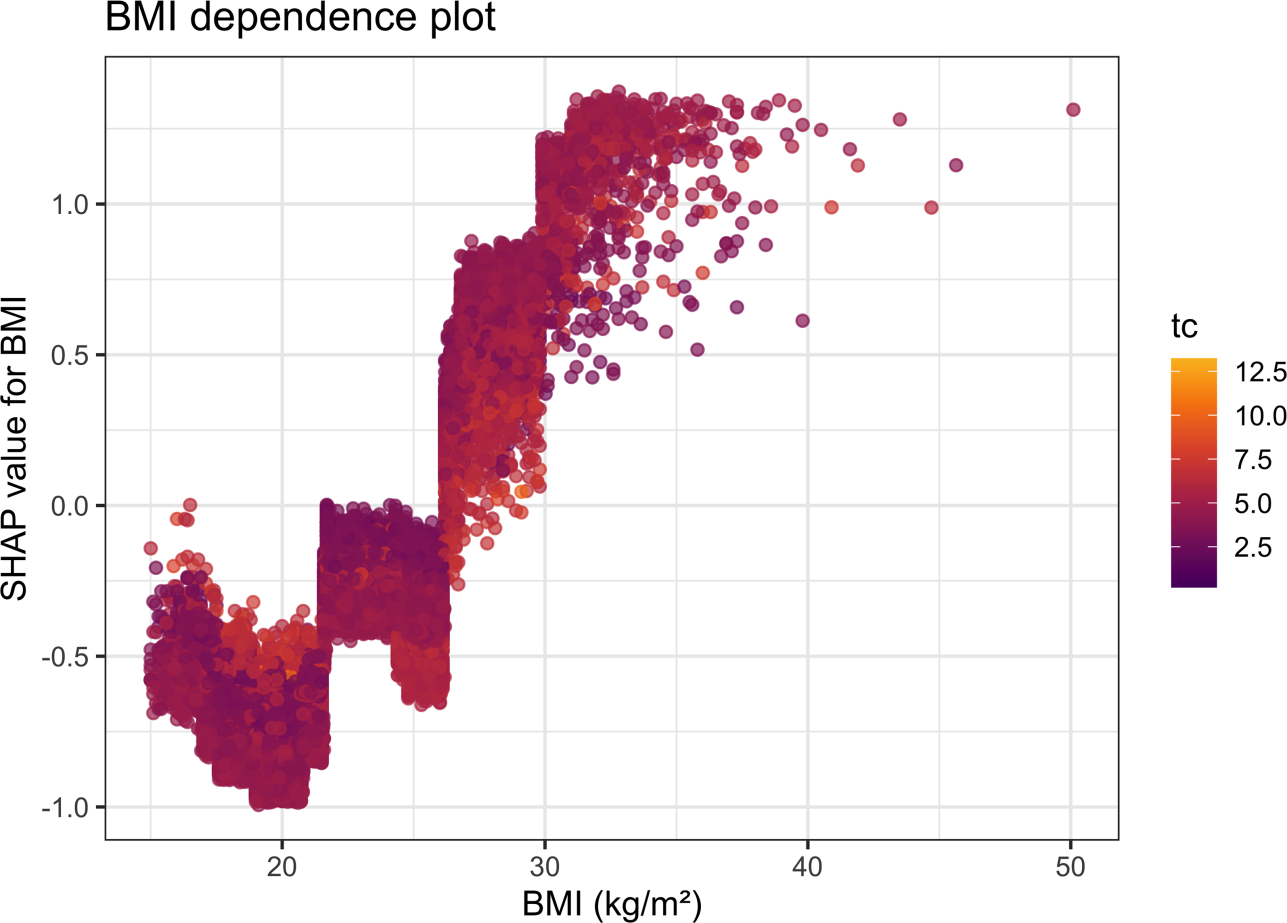
SHAP dependence plot for BMI.

The BMI dependence plot in **Figure 6** further showed an evident nonlinear contribution of BMI to model output: at lower BMI levels, SHAP values were mainly negative; when BMI increased to approximately 25 kg/m², its contribution gradually became positive; and after approximately 28–30 kg/m², the increase became more pronounced, indicating that beyond this range, BMI substantially amplified future diabetes risk. This result further supports that the relationship between BMI and diabetes risk is not simply linear but shows a more marked risk-amplifying effect at higher BMI levels.

Further SHAP interaction visualizations suggested possible model-derived joint contribution patterns between BMI and triglycerides and between BMI and SBP in the exploratory XGBoost model, whereas no clear model-derived interaction pattern was observed between BMI and family history of diabetes. However, these visualizations should not be interpreted as evidence of formal statistical interaction. Instead, they indicate how the trained XGBoost model internally represented nonlinear contribution patterns across combinations of predictors. Therefore, the SHAP interaction findings were regarded as exploratory and hypothesis-generating and were further examined using prespecified Cox-based interaction tests. It should be noted that although total cholesterol ranked highly in the SHAP importance analysis, its association did not reach statistical significance in the multivariable Cox model; therefore, it is more appropriate to describe it as a variable with high contribution in the exploratory machine learning model rather than as an independent risk factor. Similarly, although family history of diabetes was not prominent in the SHAP global contribution ranking or interaction structure, it remained a significant independent correlate in the multivariable Cox model. This suggests that Cox regression primarily reflects the average independent association of variables with the outcome, whereas SHAP mainly captures the global contribution pattern and interaction structure within the XGBoost model. In addition, the low prevalence of a positive family history may also have contributed to its relatively small average SHAP value.

### 3.6 Formal testing of BMI-related high-risk feature combinations

**Figure 7:**
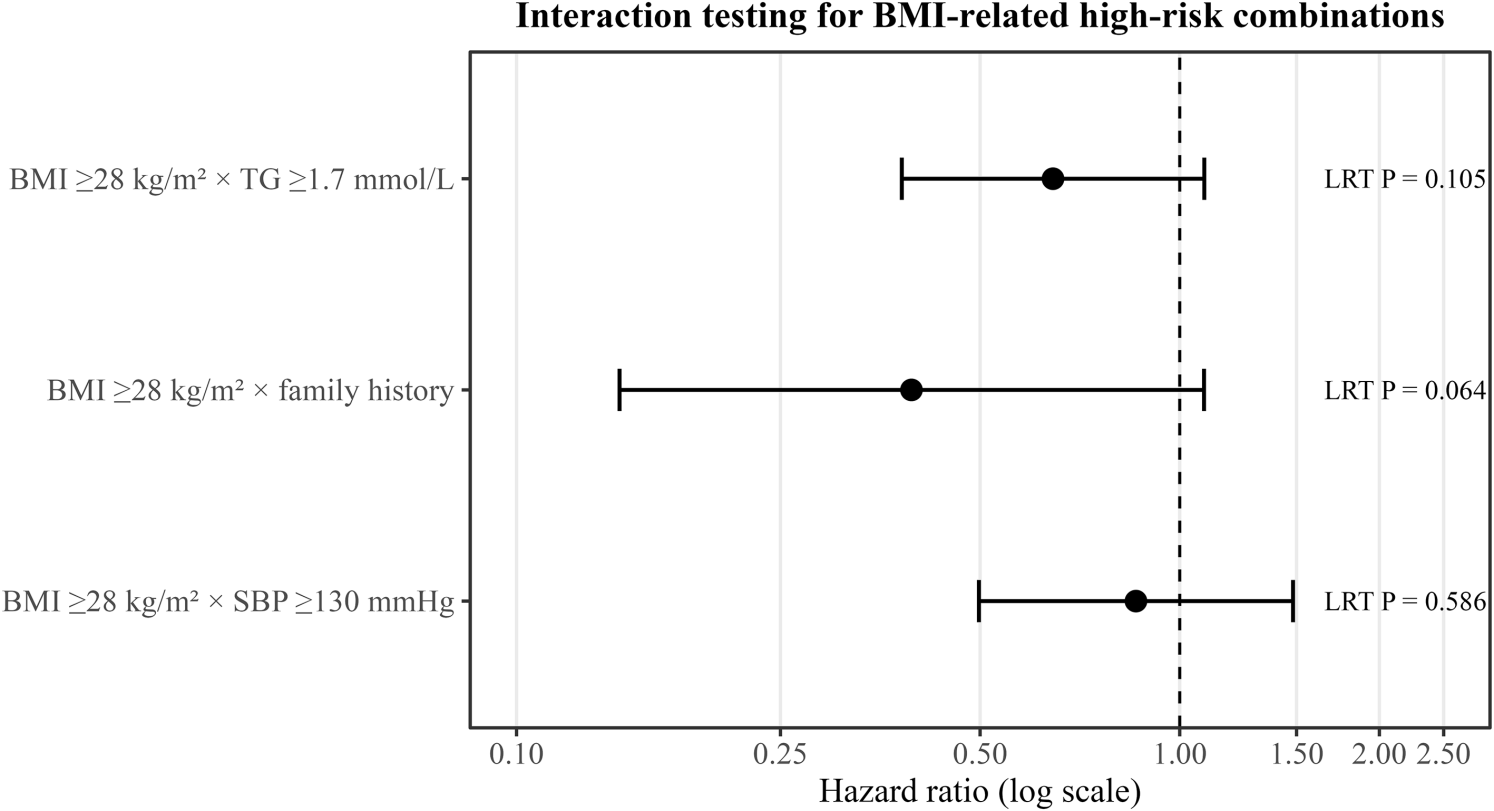
SHAP interaction visualization between BMI and key variables.

To formally examine whether the exploratory SHAP-derived joint contribution patterns corresponded to statistical interactions, Cox proportional hazards regression was used to test prespecified threshold-based interaction terms between BMI and related clinical variables (**Table 5**; **Figure 7**). Because both the RCS and SHAP analyses suggested that the risk effect of BMI became more pronounced in the higher range, threshold-based variables were used to construct interaction terms. High BMI was defined as BMI ≥28 kg/m², high triglycerides as TG ≥1.7 mmol/L, and high SBP as SBP ≥130 mmHg. Interactions between high BMI and triglycerides, family history of diabetes, and SBP were examined separately. The BMI ≥28 × family history of diabetes interaction term showed a borderline trend but did not reach conventional statistical significance (HR = 0.394, 95% CI: 0.143–1.088, P for interaction = 0.064). Given the very low number of incident diabetes events, the statistical power for interaction analyses was limited. Therefore, this borderline finding should be interpreted cautiously and should not be considered evidence of a confirmed modifying effect. Similarly, the BMI ≥28 × TG ≥1.7 mmol/L and BMI ≥28 × SBP ≥130 mmHg interaction terms did not reach statistical significance. Overall, the Cox-based interaction analyses did not provide definitive evidence of multiplicative interactions between high BMI and family history, triglycerides, or systolic blood pressure. The BMI ≥28 × TG ≥1.7 mmol/L interaction term did not reach statistical significance but also showed a suggestive trend (HR = 0.644, 95% CI: 0.381–1.089, P for interaction = 0.105). By contrast, the BMI ≥28 × SBP ≥130 mmHg interaction term was not significant (HR = 0.859, 95% CI: 0.498–1.482, P for interaction = 0.586). Overall, the formal threshold-based interaction tests did not identify definitive statistically significant interactions, but they suggested a possible joint risk pattern between high BMI and family history of diabetes, while the joint effect of high BMI and elevated triglycerides warrants further investigation in larger samples.

### 3.7 Internal validation of the LR primary model

**Figure 8:**
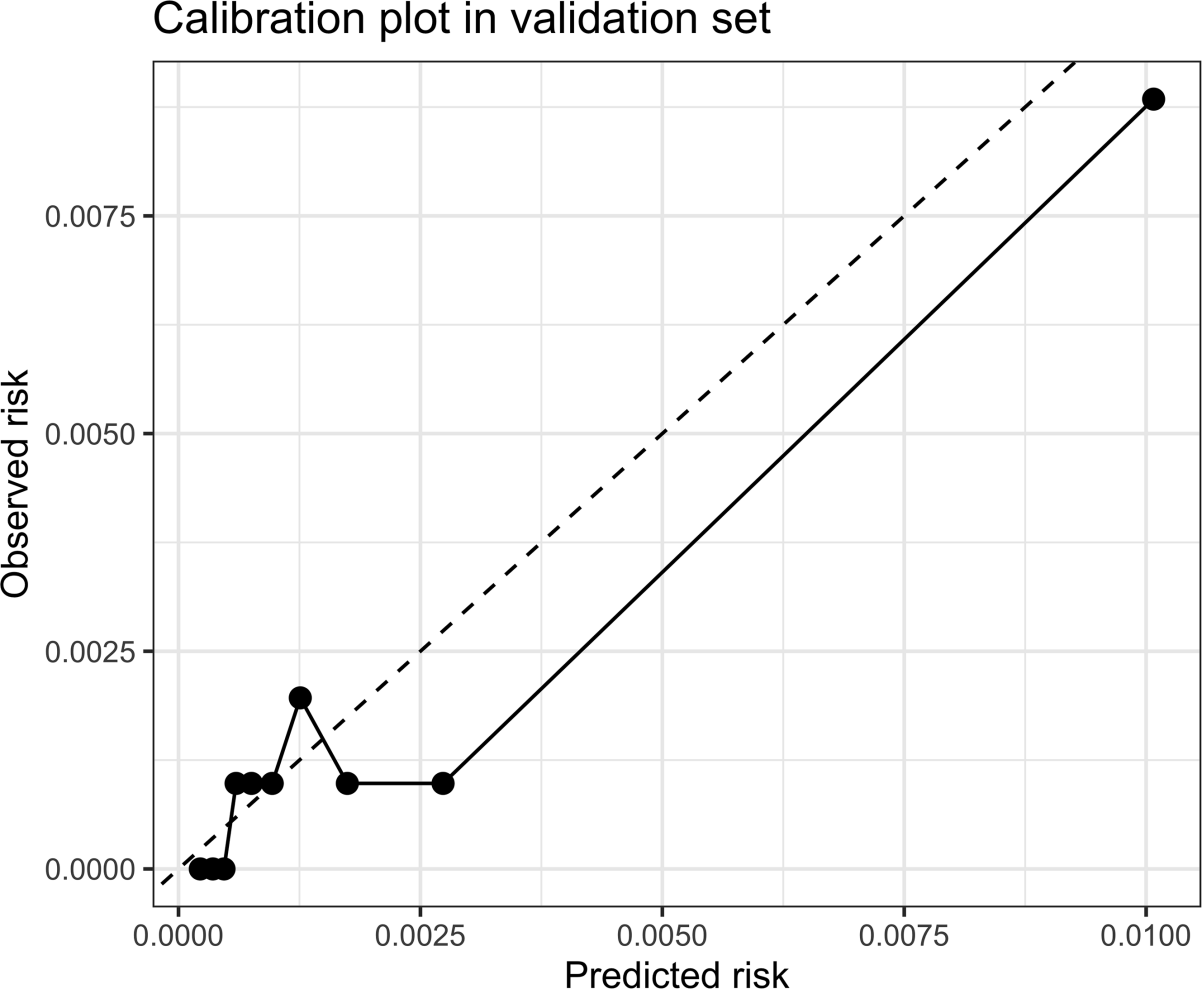
Calibration curve of the LR primary model in the validation set.

**Figure 9:**
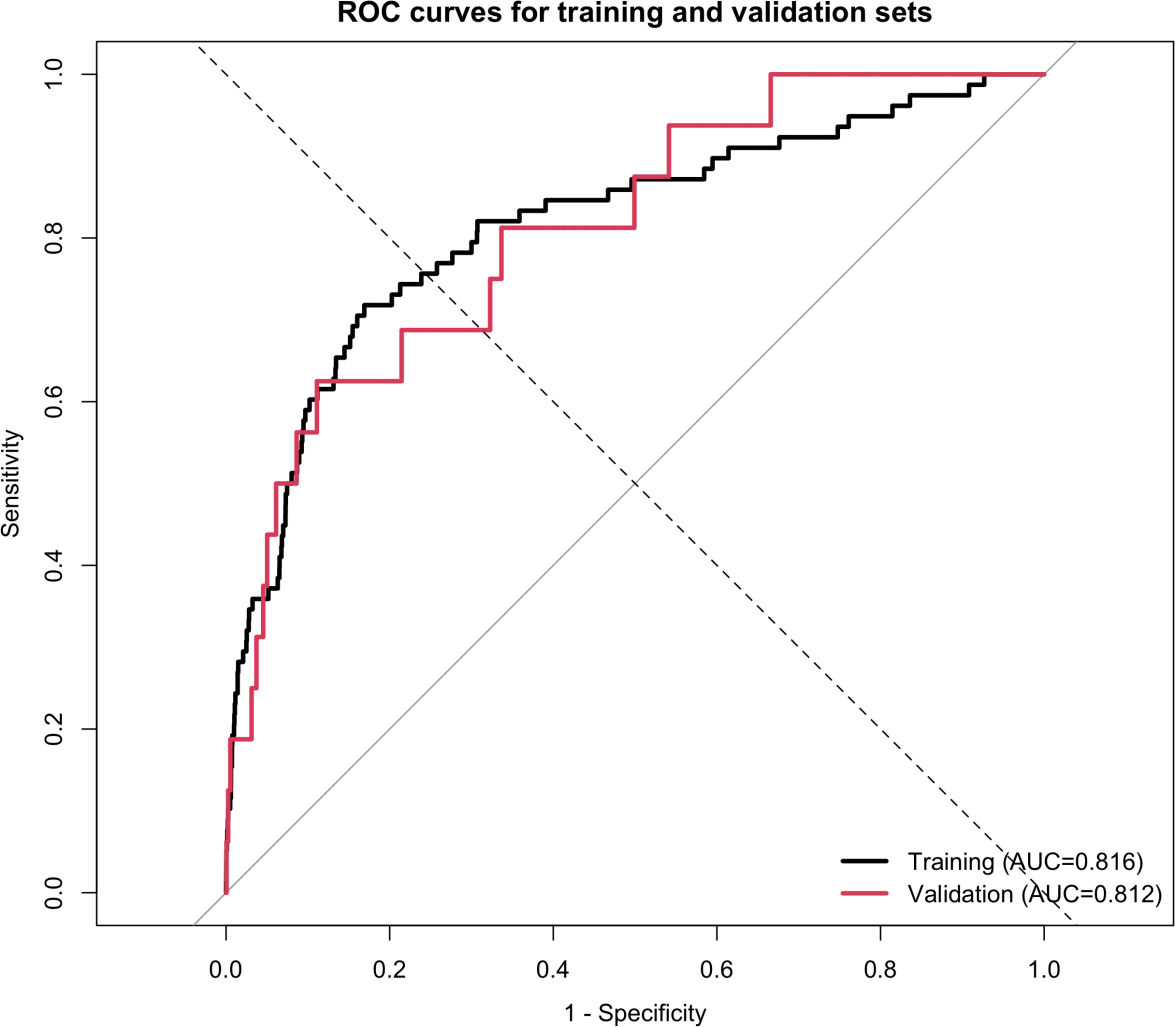
ROC curves of the LR primary model in the training and validation sets.

The internal validation results of the LR primary model are presented in **Table 4**, and the ROC curves and calibration plots are shown in **Figures 8** and **9**, respectively. The AUCs in the training and validation sets were 0.816 and 0.812, respectively, indicating good discrimination in both datasets and stable generalizability without obvious overfitting. The Brier score was 0.002 in both datasets, however, given the very low event rate, this value should be interpreted together with PR-AUC and other classification metrics rather than as standalone evidence of strong predictive performance.

**Table 4:** Comparison of model performance.

Model	Dataset	AUC	PR-AUC	Sensitivity	Specificity	PPV	NPV	Brier score
LR	Training	0.816	0.037	0.718	0.831	0.008	0.999	0.002
CART	Training	0.500	0.002	1	0	0.002		0.002
RF	Training	1.000	1.000	1	1	1	1	0.003
XGBoost	Training	0.871	0.023	0.821	0.763	0.007	1	0.22
LR	Validation	0.812	0.017	0.625	0.832	0.006	0.999	0.002
CART	Validation	0.500	0.002	1	0	0.002		0.002
RF	Validation	0.673	0.005	0	1		0.998	0.004
XGBoost	Validation	0.817	0.009	0.688	0.768	0.005	0.999	0.22

**Table 5:** BMI-related high-risk feature combinations.

Interaction	Term	HR (95% CI)	Wald P	LRT P	N	Events
BMI ≥28 × TG ≥1.7	bmi28:tg_high	0.644 (0.381, 1.089)	0.101	0.105	100929	258
BMI ≥28 × family history	bmi28:fhx_dm_num	0.394 (0.143, 1.088)	0.072	0.064	100929	258
BMI ≥28 × SBP ≥130	bmi28:sbp130	0.859 (0.498, 1.482)	0.584	0.586	100929	258

The PR-AUC values were 0.037 and 0.017 in the training and validation sets, respectively. These low PR-AUC values indicate that, despite acceptable ROC-AUC values, the model had limited ability to identify true positive cases under the highly imbalanced outcome distribution. Therefore, the LR primary model should be interpreted as a preliminary tool for short-term 3-year diabetes risk stratification among young adults with normal fasting glucose, rather than as a standalone clinical prediction or decision-making model. Overall, the model may help distinguish relatively higher-risk individuals at the population level, but its clinical utility for directly identifying future diabetes cases remains limited and requires further validation.

## 4 Discussion

This study focused on young adults with normal baseline fasting plasma glucose. The main findings were as follows. First, BMI remained independently associated with incident diabetes in this population, suggesting that even before the threshold for abnormal fasting glucose is reached, individuals with higher BMI may already be at an early stage of increased future diabetes risk. Second, the relationship between BMI and diabetes risk was not simply linear; rather, a more pronounced risk-amplifying effect was observed at higher BMI levels. Third, taken together, the SHAP analysis and formal interaction testing suggested that BMI-related risk is not isolated, but is more likely embedded within a metabolic risk structure jointly shaped by blood lipids, blood pressure, and family-related factors.

The most central finding of this study is that, among young adults with baseline FPG <5.6 mmol/L, BMI still provided an independent signal for future diabetes risk. This suggests that diabetes risk identification in young adults should not be limited to whether fasting glucose has already reached the abnormal or diagnostic range, because some individuals may already be on a sustained progression pathway driven by overweight, obesity, and related metabolic abnormalities despite remaining normoglycemic. Furthermore, both the RCS and SHAP analyses showed that the effect of BMI on diabetes risk did not increase uniformly, but became more pronounced at higher BMI levels, especially beyond approximately 28 kg/m². These findings indicate that it may be insufficient to interpret the relationship between BMI and diabetes risk solely as a single linear effect and that the higher BMI range in young adults may correspond to a stronger amplification of metabolic risk.

Beyond BMI itself, the present study further suggests that BMI-related risk may be better understood as a metabolic high-risk structure centered on weight status and accompanied by lipid and blood pressure abnormalities. SHAP analysis showed that BMI ranked first in the exploratory XGBoost model and exhibited some degree of joint patterning with triglycerides and SBP, suggesting that future diabetes risk may deserve greater attention when high BMI coexists with elevated TG and/or elevated blood pressure. It should be noted, however, that the formal multiplicative interaction tests based on Cox regression did not provide clear statistical support, with only the BMI ≥28 × family history term showing a borderline trend. This can be interpreted in two ways. On the one hand, SHAP reflects the global contribution pattern and complex nonlinear structure within a machine learning model, whereas traditional Cox interaction testing targets a pre-specified multiplicative interaction form; the two therefore do not focus on exactly the same aspect. On the other hand, the relatively limited number of outcome events in the present study may also have reduced the statistical power of the interaction analyses. Accordingly, the current findings are better suited to supporting the overall concept of a BMI-related high-risk structure, rather than being overinterpreted as fully confirmed strong interactions.

Previous studies have shown that young-onset type 2 diabetes is associated with higher risks of all-cause mortality, cardiovascular complications, and microvascular complications, underscoring the importance of identifying high-risk individuals during young adulthood (3). Prior research has also suggested that BMI is an important indicator for diabetes risk identification, but existing risk prediction models generally require the integration of multiple factors, such as age, family history, blood pressure, waist circumference, and fasting glucose, for comprehensive stratification (15). Building on this evidence, the present study further restricted the analysis to young adults with normal fasting glucose and combined conventional regression with interpretable machine learning, thereby not only confirming the independent association of BMI but also demonstrating its nonlinear risk pattern and potential joint risk structure. Compared with studies focusing only on the average effect of a single risk factor, this study more clearly highlights the heterogeneity of BMI-related risk and emphasizes the clinically important reality that “normal glucose does not necessarily mean low risk” in young adults.

The findings of this study also have potential clinical and public health implications. First, among young adults undergoing routine health examinations, normal fasting glucose should not be interpreted as negligible future diabetes risk, especially among those with higher BMI, in whom the accumulation of metabolic abnormalities may already be underway. Second, BMI, as a simple and easily obtainable indicator, may serve as an initial entry point for diabetes risk identification in young adults; however, a more reasonable approach is to interpret it together with blood lipids, blood pressure, and family history. Third, the critical high-BMI range suggested by this study may have practical value for early screening, intensified follow-up, and stratified lifestyle intervention in young adults. In other words, this analytical framework may help identify young individuals who are easily overlooked under traditional glycemic criteria but who, in fact, already carry a relatively high future risk. However, the time horizon of the present findings should be interpreted carefully. The median follow-up duration was 2.99 years, which is relatively short for diabetes development in a young normoglycemic population. Thus, the observed associations and prediction results mainly reflect short-term 3-year diabetes risk rather than long-term cumulative risk. With longer follow-up, more incident events may occur, and the relative importance of predictors, the apparent BMI risk threshold, and model performance may change. Therefore, the current models should not be directly extrapolated to long-term diabetes prediction without further validation.

This study has several strengths. First, it focused on young adults with normal fasting glucose, a population of high relevance for primary diabetes prevention but one that has been relatively understudied. Second, by combining conventional statistical methods with interpretable machine learning, the study was able not only to evaluate the average association between BMI and incident diabetes but also to further uncover its nonlinear relationship and potential high-risk feature combinations. Third, the Cox, RCS, SHAP, and internal validation results showed good directional consistency, lending support to the study conclusions from multiple analytical perspectives.

Several limitations should also be acknowledged. First, this study was based on a single data source, and the participants were mainly derived from a health check-up population; thus, the generalizability of the findings should be interpreted with caution. Second, only internal validation has been completed, and further validation in independent external cohorts is still needed. Third, the number of incident diabetes events was limited despite the large overall sample size. This low event rate led to low PR-AUC and PPV values, indicating limited ability of the models to identify true positive cases in clinical practice. It also reduced the statistical power of the interaction analyses, making borderline interaction findings difficult to interpret. Therefore, the interaction-related results should be regarded as exploratory and hypothesis-generating rather than confirmatory. Fourth, the median follow-up duration was relatively short for capturing diabetes incidence in young adults with normal fasting glucose. This may have limited the number of observed events and restricted the ability to characterize the long-term trajectory of risk accumulation. Therefore, the findings should not be extrapolated directly to long-term diabetes prediction without further validation.

## 5 Conclusion

This study combined Cox regression, restricted cubic spline analysis, machine learning, and SHAP interpretation to identify BMI-related residual diabetes risk among young adults with normal baseline FPG. BMI was independently associated with incident diabetes and showed a nonlinear risk pattern, with risk increasing more clearly around and above 28 kg/m². LR and XGBoost showed stable validation performance, and SHAP interpretation of the exploratory XGBoost model suggested possible model-derived joint contribution patterns involving BMI, triglycerides, and blood pressure; however, these patterns were not confirmed by formal Cox-based interaction testing. Therefore, the interaction-related findings should be interpreted as exploratory and require confirmation in cohorts with more outcome events and longer follow-up. Compared with conventional approaches based mainly on glycemic status or single risk factors, this framework may support earlier and more refined diabetes risk identification in normoglycemic young adults.

## Data Availability

The original contributions presented in the study are included in the article/**Supplementary Material**. Further inquiries can be directed to the corresponding authors.
